# Robustness of the healthcare utilization results from the Rotavirus Efficacy and Safety Trial (REST) evaluating the human-bovine (WC3) reassortant pentavalent rotavirus vaccine (RV5)

**DOI:** 10.1186/1471-2431-10-42

**Published:** 2010-06-11

**Authors:** Robbin Itzler, Gary Koch, David O Matson, Leif Gothefors, Pierre Van Damme, Mark J DiNubile, Penny M Heaton

**Affiliations:** 1 Merck Research Laboratories, West Point, Pennsylvania, USA; 2 University of North Carolina, Chapel Hill, North Carolina, USA; 3 Eastern Virginia Medical School and Old Dominion University, Norfolk, Virginia, USA; 4 University of Umeå, Umeå, Sweden; 5 University of Antwerp, Antwerp, Belgium; 6 Novartis, Cambridge, MA, USA

## Abstract

**Background:**

The Rotavirus Efficacy and Safety Trial was a placebo-controlled Phase III study that evaluated the safety and efficacy of a three-dose pentavalent rotavirus vaccine (RV5) including its effect on healthcare utilization for rotavirus gastroenteritis (RVGE). The per-protocol (PP) analyses, which counted events occurring 14 days after dose 3 among infants without protocol violations, have already been published. This paper evaluates the consistency of the healthcare utilization results based on the modified intention to treat (MITT) analyses with the PP analyses. The MITT analyses include all infants receiving at least one dose of vaccine or placebo and follow-up begins after dose 1. The paper also explores the consistency of the results for different subgroups of the study population with different types of surveillance.

**Methods:**

Data on healthcare utilization for acute gastroenteritis were collected via telephone interviews after administration of the first dose. Parents were either contacted every 6 weeks or every 2 weeks depending on the substudy in which they were enrolled. Those contacted every 2 weeks were also asked to complete symptom diaries. Poisson regression was used to evaluate the effect of RV5 on the rates of RVGE-associated healthcare encounters in all of the analyses.

**Results:**

In the first 2 years after vaccination, RV5 reduced the combined rate of hospitalizations and emergency department (ED) visits 88.9% (95% CI: 84.9, 91.9) for all RVGE regardless of serotype in the MITT analysis compared with a 94.5% (95% CI: 91.2, 96.6) reduction based on the G1-G4 PP analysis. By type of surveillance, the rate reductions for the G1-G4 PP analysis were 91.0% (95% CI: 81.7, 95.5) and 95.9% (95% CI: 92.2, 97.8) among parents contacted every 2 weeks (number evaluable = 4,451) and every 6 weeks (number evaluable = 52,683) respectively.

**Conclusions:**

Our analyses demonstrated that the effect of RV5 on reducing the rate of hospitalizations and ED visits based on the MITT analyses were generally consistent with the PP analyses. The rate of events for subgroups with different intensities of surveillance differed but the effect of RV5 on the relative rate reductions were consistent with the results that have already been published.

**Trial Registration:**

ClinicalTrials.gov number, NCT00090233

## Background

Prior to the introduction of rotavirus vaccines, rotavirus gastroenteritis (RVGE) was the most common cause of severe gastroenteritis in infants and young children. Almost every child has been infected with rotavirus by 5 years of age in both developing and industrialized countries [1]. Rotavirus-positive acute gastroenteritis is more severe than rotavirus-negative acute gastroenteritis with higher rates of dehydration, vomiting, fever, and signs of lethargy [2]. There are nearly 600,000 deaths due to RVGE worldwide which mainly occur in developing countries [1]. In industrialized countries, hospitalization rates had been high prior to the introduction of vaccination programs [3]. Children are usually admitted to the hospital to prevent or treat dehydration [4-6]. RVGE most often occurs at specific times of the year [7]. In the United States, for example, rotavirus activity follows a distinct winter-spring seasonal pattern [8].

Two rotavirus vaccines are currently being marketed in various countries throughout the world to prevent RVGE. One is a monovalent G1P1A[8] human rotavirus vaccine (RV1; Rotarix^®^, GlaxoSmithKline Biologicals, Rixensart, Belgium). The other is a pentavalent (G1, G2, G3, G4, P1A[8]) human bovine (WC3 strain) reassortant rotavirus vaccine (RV5; RotaTeq^®^, Merck & Co., Inc., Whitehouse Station, NJ, USA) [9]. Both vaccines were found to be safe and efficacious in large studies powered to detect intussusception risk [10-13]. Both vaccines have also been recommended for routine immunization in the U.S. as well as several countries in Latin America, Europe, and Australia [14].

Since RV5 was included in the immunization schedule in the U.S., surveillance studies have been conducted to evaluate vaccine effectiveness. In a national sentinel network of laboratories, the median length of the rotavirus season decreased by 54%, the number of stool specimens submitted for testing decreased by 67%, and the proportion of test results positive for rotavirus declined by 69% after vaccination in the 2007-8 season compared to the 2000-2006 seasons [15]. Similar results were reported at Children's Hospital of Philadelphia [16]. In the 2007-8 seasons, there was an 87% decline in community acquired rotavirus cases compared to the same time period in 2005-2006. Other studies reported sharp reductions in rotavirus-related hospitalizations and emergency department (ED) visits after RV5 became available even among age groups that would not have been eligible to receive the vaccine [17-19]. These reductions in healthcare utilization parallel what was observed in the Phase III trial.

The efficacy and safety of RV5 was evaluated in the Rotavirus Efficacy and Safety Trial (REST), a large international placebo-controlled, blinded trial conducted from 2001 to 2005 with nearly 70,000 healthy infants enrolled from 11 countries. Infants were to receive three doses of either RV5 or placebo. The large sample size and the length of follow-up in REST provided a unique opportunity to quantify the effect of RV5 on healthcare outcomes related to RVGE in a prelicensure study. In the per-protocol (PP) analysis, which was comprised of infants without protocol violations and counted events occurring 14 days after dose 3, RV5 reduced the rate of hospitalizations and ED visits related to G1-G4 RVGE by 94.5% (95% CI: 91.2 - 96.6) in the first two years after vaccination [10]. The rate reductions were generally consistent among study populations across three regions - the US (including the Navajo and White Mountain Apache Nations), Europe, and Latin America [20].

The efficacy reported in clinical trials can not always be directly extrapolated to other populations because of the strict inclusion criteria for enrollment as well as the close monitoring of subjects in the trial [21,22], especially when the results are based on a PP analysis. This paper assesses the robustness of the previously published RVGE-associated healthcare utilization results from REST based on the PP analysis by evaluating the reduction in RVGE-associated healthcare utilization regardless of serotype in a modified intention-to-treat (MITT) analysis among infants receiving at least one dose of vaccine. The method and frequency of surveillance also differed for specific subgroups within the study population. The effects of the differences in intensity of surveillance on healthcare utilization rates were also explored.

## Methods

### Study design and case definitions

In REST, infants were randomly assigned in a 1:1 ratio to receive either vaccine or visibly indistinguishable placebo starting at 6-12 weeks of age, followed by two more doses at 4-10 week intervals up to 32 weeks of age [10,20]. Doses were administered year-round without regard to RV seasonality. The case definition for acute gastroenteritis (AGE) was 3 or more watery or looser-than-normal stools within a 24-hour period and/or forceful vomiting along with the identification of rotavirus by enzyme immunoassay (EIA) in a stool specimen taken within 14 days after the onset of symptoms. G-serotypes were identified by one-step reverse-transcriptase-polymerase chain reaction (PCR) amplification followed by sequencing [23]. More complete detail about the study design and the methods used to analyze the data were provided in the earlier publications [10,20]. The protocol was approved by the ethical review committee at each site and conducted in conformance with applicable country or local requirements. All guardians signed informed consent prior to vaccination.

### PP vs. MITT analyses

Earlier publications have presented the PP analyses for the effect of RV5 on reducing the rates of hospitalizations and/or ED visits for the overall study population as well as the rate reductions by region and serotype for the first two years after vaccination. The analyses for the overall study population and the analyses stratified by region were based on the G1, G2, G3, and G4 serotypes included in RV5. The effect of RV5 on hospitalizations and ED visits combined was also determined individually for each of the 5 most common G-serotypes circulating in Europe and the Americas: G1, G2, G3, G4 and G9.

For the analyses stratified by region, three separate regions were examined: the US including the Navajo and White Mountain Apache Nations (but not Puerto Rico), Europe, and Latin America or the Caribbean including the Commonwealth of Puerto Rico [20]. Although a self-governing island that is part of the commonwealth of the United States, Puerto Rico was included in the Latin American/Caribbean region for these analyses because of its close proximity to the Caribbean.

In this paper we compare the G1-G4 PP analyses with the MITT analyses regardless of serotype based on infants receiving at least one dose of vaccine or placebo where all healthcare encounters anytime after the first dose are counted for the first two years after vaccination. The analyses for the overall study population as well as the analyses stratified by region include all episodes of RVGE identified by EIA regardless of G-serotypes including episodes where rotavirus antigen was detected by enzyme immunoassay but the serotype could not be determined by PCR. Table 1 provides a detailed comparison of the inclusion criteria for the PP and MITT analyses. Other variations of the PP and MITT analyses were also presented in order to assess the potential effect of including all RVGE serotypes, subjects with protocol violations, and the protection prior to completion of the vaccine regimen for the overall study population.

**Table 1:** Comparison of Inclusion/Exclusion Criteria for the Per- Protocol (PP) and Modified Intention-to-Treat (MITT) Analyses

Criteria for Comparison	PP Analysis	MITT Analysis
		

Infants Randomized but Not Vaccinated^a^	Excluded	Excluded

		

Infants without Follow-up	Excludes infants where the end of follow up occurs before 14 days after dose 3	Excludes infants where the end of follow up is the same day as the first vaccination^a^

		

Infants classified as not-evaluable (Infants are classified as not-evaluable if they have one or more episodes not-evaluable for rotavirus and no episodes positive for rotavirus)	Episodes were classified as not-evaluable if the stool specimen was positive for wild-type rotavirus prior to 14 days after dose 3, if there was incomplete clinical and/or laboratory results, or the stool specimen was collected outside the 14 day range after symptom onset	Episodes were classified as not-evaluable if the clinical and/or laboratory results were incomplete or the stool specimen was collected outside the 14 day range after symptom onset

		

Infants with protocol violations	Excluded	Included

		

Serotypes	Overall analysis was limited to RVGE due to G1-G4 serotypes	Overall analysis included all RVGE regardless of serotype

^a ^There were no healthcare encounters on the day of the first vaccination.

### Method and frequency of surveillance

Several substudies were nested within REST. The surveillance method and the frequency of contact with parents differed between the Clinical Efficacy substudy and the remainder of the REST cohort. Data on healthcare outcomes was collected via telephone interviews with parents for all infants. Parents were contacted on days 7, 14, and 42 after each dose. The parents of infants in the Clinical Efficacy substudy were then contacted every two weeks until their children completed the study whereas the parents of the infants not in the substudy were contacted every 6 weeks. Health Care Utilization Questionnaires were completed by study personnel during the interviews. For infants participating at sites included in the substudy, all episodes of AGE were reported and parents completed a daily Acute Gastroenteritis Report Card which permitted an assessment of the severity of each acute gastroenteritis episode. For infants not included in the Clinical Efficacy substudy, only hospitalizations and ED visits associated with episodes of AGE were reported and no diary cards were completed. This paper also explores how the intensity of surveillance affected the reported rates of healthcare encounters for RVGE and the observed relative reduction in the rate of RVGE healthcare encounters by comparing the results at study sites included in the Clinical Efficacy substudy with all other sites.

Given that there were some AGE episodes where stool specimens were not collected, all AGE episodes were also analyzed to determine whether any differences between the two subgroups with respect to AGE/RVGE between subjects enrolled in the Clinical Efficacy substudy and other subjects in REST were due to differences in the rates of healthcare encounters for AGE reported or differences in the ability to collect stool specimens and confirm whether the encounters were associated with RVGE.

### Statistical analyses

Poisson regression was used to compare the rates of healthcare encounters in the vaccine and placebo groups. For the analyses based on the overall study population and by serotype [10], generalized estimating equations (GEE) were used to adjust the standard errors and account for correlations between observations [24,25]. GEE is not optimal when the count of events is small [26]. However, because this methodology was applied to the serotype specific PP analyses in an earlier publication, it is applied here to the MITT analyses to be consistent. GEE also cannot be used when there are no healthcare encounters in the vaccine or placebo groups. Therefore, the exact binomial method for ratios of Poisson counts was used for the analyses stratified by region as well as for analyses where there was a zero count in one of the groups being compared. The rates of healthcare encounters were expressed as the annual number of encounters per 1000 person-years because the length of follow-up differed among infants enrolled in the study. All infants were to be followed for at least 42 days after the last vaccination and up to 365 days after the first dose or until the end of the study, whichever came first. A subset of infants in the Clinical Efficacy substudy enrolled during a rotavirus season was followed until the end of the next rotavirus season which resulted in follow-up for time periods for a maximum of 2 years for some infants.

## Results

### Infants included in the MITT and PP analyses

**Figure 1:**
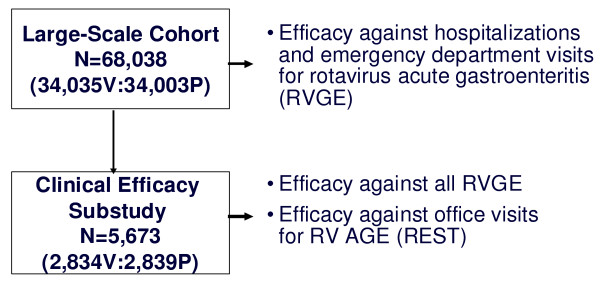
**REST Study Design with Respect to the Collection of the Healthcare Utilization Data**. N = number vaccinated.

Data for 69,274 randomly assigned infants were available in the REST clinical database at the time the Data and Safety Monitoring Board determined that the study had satisfied its criterion for stopping enrollment associated with the primary safety endpoint assessing the risk of intussception among vaccine recipients. After that date, an additional 1027 infants were only followed for safety but not efficacy. In total, 68,038 infants received at least one dose of vaccine or placebo including 5,673 vaccinated infants in the efficacy substudy (Figure 1). The PP analyses included 28,646 evaluable infants in the RV5 group and 28,488 evaluable infants in the placebo group. There were 9,518 infants (4,740 RV5; 4,778 Placebo) with protocol violations and 8,773 infants (4,367 RV5; 4,406 Placebo) violated the protocol because they did not receive all 3 doses.

**Figure 2:**
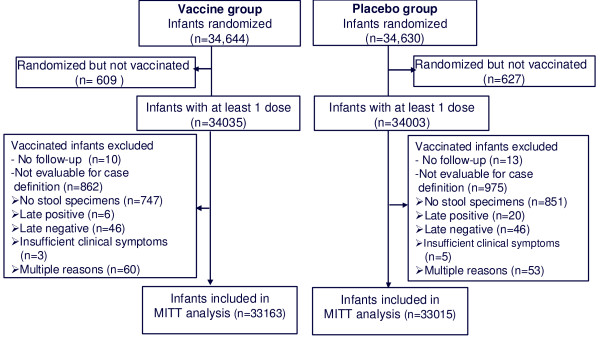
**Subject Accounting for MITT Analyses**. There were no health care encounters on the first day of vaccination among 26 infants without follow-up after the first day of vaccination.

The MITT analysis included 33,163 evaluable infants in the RV5 group and 33,015 evaluable infants in the placebo group. The MITT analysis differed from a strict intention-to-treat (ITT) population because it excluded infants who were randomized but never vaccinated since these infants were not followed for efficacy outcomes. Figure 2 accounts for all of the infants excluded from the MITT analysis in the RV5 and placebo groups with the reasons for exclusion. Only 1.8% of the infants randomized were excluded from the MITT analysis because they were never vaccinated. Of the 1,860 infants receiving at least one dose of vaccine or placebo excluded from the MITT analysis, 92.3% reported an episode of acute gastroenteritis but either no stool specimens were collected to determine whether they were positive for rotavirus or the stool specimens were collected outside the allowed time period. Table 2 provides a comparison of the baseline characteristics for age, race, and gender based on the number of evaluable subjects for the G1-G4 PP analysis and the MITT analysis regardless of serotype.

**Table 2:** Comparison of Baseline Characteristics for PP and MITT Analyses based on the Number of Evaluable Subjects

	PP Analysis		MITT Analysis	
	**RV5**	**Placebo**	**RV5**	**Placebo**

*Infants vaccinated*	34035	34003	34035	34003

*Protocol violators*^*a*^	4740	4778	****	****

*Infants with no follow-up*^*b*^	26	25	10	13

*Infants classified as not-evaluable*	623	712	862	975

*Infants contributing to the analysis*	28646	28488	33163	33015

				

Age at Entry - in weeks				

Mean	9.8	9.8	9.8	9.8

Median	10	10	10	10

Range	(6-13)	(6-13)	(6-13)	(6-13)

				

Race - number, %				

White	20676 (72.2%)	20653 (72.5%)	23021 (69.4%)	22912 (69.4%)

Black	2230 (7.8%)	2215 (7.8%)	2739 (8.3%)	2761 (8.4%)

Hispanic	3644 (12.7%)	3552 (12.5%)	4721 (14.2%)	4656 (14.1%)

Other	2096 (7.3%)	2068 (7.2%)	2682 (8.1%)	2686 (8.1%)

				

Gender - number, %				

Female	14112 (49.3%)	14131 (49.6%)	16343 (49.3%)	16331 (49.5%)

Male	14534 (50.7%)	14357 (50.4%)	16820 (50.7%)	16684 (50.5%)

^a ^Subjects who received a vial where a temperature excursion occurred, subjects who had less than 3 vaccinations or less than 28 days between vaccinations, and subjects who were cross-treated or prematurely unblinded.

^b ^In the PP analysis follow-up begins 14 days after dose 3; in the MITT analysis follow-up begins after dose 1

### Comparison of results based on PP analysis for serotypes G1-G4 and PP analysis regardless of serotype for overall study population

There were 389 hospitalizations and emergency department (ED) visits (20 RV5; 369 Placebo) in the PP analysis limited to the G1-G4 serotypes (Table 3). When all episodes of rotavirus gastroenteritis regardless of serotype were included in the PP analysis (with follow-up beginning 14 days after administration of the third dose), there were 429 hospitalizations and ED visits (22 RV5, 407 P - data not shown in tables) and RV5 reduced the rate of hospitalizations and ED visits for the first 2 years after vaccination by 94.6% (95% CI: 91.5, 96.5) for the PP analysis regardless of serotype compared with a 94.5% (95% CI: 91.2, 96.6) rate reduction based on the PP analysis for the G1-G4 serotypes. In addition to the 389 hospitalizations and ED visits included in the PP analysis limited to the G1-G4 serotypes, the PP analysis regardless of serotype also included 14 (0 RV5, 14 P) hospitalizations and ED visits associated with the G9 serotype, 1 ED visit associated with the G12 serotype, and 25 (2 RV5, 23 P) hospitalizations and ED visits where the serotype could not be identified by PCR.

**Table 3:** Comparison of PP and MITT Analyses for RVGE Healthcare Encounters by Type of Encounter

	PP Analysis			MITT Analysis		
	**RV5**	**Placebo**	**% Rate Reduction** **(95% CI)**	**RV5**	**Placebo**	**% Rate Reduction** **(95% CI)**

**Hospitalizations and ED Visits**						

*Infants vaccinated*	34035	34003		34035	34003	

*Protocol violators*^*a*^	4740	4778		****	****	

*Infants with no follow-up*^*b*^	26	25		10	13	

*Infants classified as not-evaluable*	623	712		862	975	

*Infants contributing to the analysis*	28646	28488		33163	33015	

No. (rate) of hospitalizations & ED visits^c^	20 (1.1)	369 (20.6)	94.5 (91.2, 96.6)	58 (2.0)	522 (18.4)	88.9 (84.9, 91.9)

No. (rate) of hospitalizations^c^	6 (0.3)	144 (8.0)	95.8 (90.5, 98.2)	16 (0.6)	215 (7.6)	92.6 (87.3, 95.7)

No. (rate) of ED visits^c^	14 (0.8)	225 (12.6)	93.7 (88.8, 96.5)	42 (1.5)	307 (10.8)	86.4 (80.2, 90.6)

**Office Visits**						

*Infants vaccinated*	2834	2839		2834	2839	

*Protocol violators*^*a*^	295	271		****	****	

*Infants with no follow-up*^*b*^	11	6		2	4	

*Infants classified as not-evaluable*	355	284		429	403	

*Infants contributing to the analysis*	2173	2278		2403	2432	

No. (rate) of office visits^c^	13 (5.5)	98 (39.7)	86.0 (73.9, 92.5)	21(6.6)	123 (38.0)	82.6 (71.6, 89.3)

^a ^Subjects who received a vial where a temperature excursion occurred, subjects who had less than 3 vaccinations or less than 28 days between vaccinations, and subjects who were cross-treated or prematurely unblinded

^b ^In the PP analysis follow-up begins 14 days after dose 3; in the MITT analysis follow-up begins after dose 1

^c ^The RV5 and Placebo columns represent the number (rate) of events; the rates reflect the incidence density expressed as the annual number of events

### Comparison of results based on PP analysis regardless of serotype and MITT analysis regardless of serotype for overall study population

When all episodes of rotavirus regardless of serotype were included and follow-up began after administration of the first dose of RV5 or placebo in the MITT analysis, there were 580 (58 RV5, 522 P) hospitalizations and ED visits included. RV5 reduced the rate of hospitalizations and ED visits by 88.9% (95% CI: 84.9, 91.9). There were 151 additional hospitalizations and ED visits in the MITT analysis compared with the PP analysis regardless of serotype. Of this total, 43 hospitalizations and ED visits (14 RV5, 29 Placebo) occurred among protocol violators and 107 hospitalizations and ED visits (21 RV5, 86 Placebo) occurred prior to 14 days after dose 3 of which 22 hospitalizations and ED visits (12 RV5, 10 Placebo) occurred within the 13 days after receiving the first dose. There was also one additional ED visit that occurred more than 14 days after dose 3 in an RV5 recipient classified as not evaluable in the PP analysis. If the MITT analysis excluded protocol violators, the reduction in the rate of hospitalizations and emergency department visits associated with RV5 would be 91.0% (95% CI: 87.3, 93.5 - data not shown in tables). If the MITT analysis excluded protocol violators and only counted RVGE-related hospitalizations and ED visits beginning 14 days after dose 1, the rate reduction would be 93.3% (95% CI: 90.2, 95.4 - data not shown in tables).

### Comparison of PP and MITT analyses stratified by serotype

The effect of RV5 on the rate of RVGE hospitalizations and ED visits combined for each of 5 common serotypes currently circulating in the Americas and Europe identified during REST are shown for the MITT and PP analyses in Table 4. Compared with the PP analysis, the number of hospitalizations and ED visits in the MITT analysis increased by 102 (16 RV5, 86 P) for the G1 serotype, 4 (0 RV5, 4 P) for the G2 serotype, 7 (2 RV5, 5 P) for the G3 serotype, 2 (0 RV5, 2 P) for the G4 serotype, and 13 (2 RV5, 11 P) for the G9 serotype. The rate reduction for the G2 serotype, 87.6% (95% CI: <0, 98.5) was not statistically significant for the PP analysis. However, the 91.7% (95% CI: 34.7, 99.0) rate reduction reflected better sensitivity of the MITT analysis to the difference between RV5 and placebo for G2. The rate reductions for the G1, G3, G4 and G9 serotypes were statistically significant based on both the PP and MITT analyses. By serotype, the rate reductions were 95.1% (95% CI: 91.6, 97.1) for G1, 93.4% (95% CI: 49.4, 99.1) for G3, 89.1% (95% CI: 52.0, 97.5) for G4, and 100% (95% CI: 69.6, 100) for G9 based on the PP analysis and 92.3% (95% CI: 88.2, 95.0) for G1, 85.1% (95% CI: 49.6, 95.6) for G3, 90.1% (95% CI: 57.2, 97.7) for G4, and 92.1% (95% CI: 66.1, 98.2) for G9 based on the MITT analysis.

**Table 4:** Comparison of PP and MITT Analyses for RVGE Hospitalizations and Emergency Department (ED) Visits by Serotype

	PP Analysis			MITT Analysis		
	**RV5**	**Placebo**	**% Rate Reduction** **(95% CI)**	**RV5**	**Placebo**	**% Rate Reduction** **(95% CI)**

*Infants vaccinated*	34035	34003		34035	34003	

*Protocol violators*^*a*^	4740	4778		****	*****	

*Infants with no follow-up*^*b*^	26	25		10	13	

*Infants classified as not-evaluable*						

G1	624	721		889	1006	

G2	628	734		904	1050	

G3	629	740		903	1062	

G4	629	740		905	1063	

G9	629	741		905	1065	

*Infants contributing to the analysis*						

G1	28645	28479		33136	32984	

G2	28641	28466		33121	32940	

G3	28640	28460		33122	32928	

G4	28640	28460		33120	32927	

G9	28640	28459		33120	32925	

*Results*^*c*^						

G1	16 (0.9)	328 (18.3)	95.1 (91.6, 97.1)	32 (1.1)	414 (14.6)	92.3 (88.2, 95.0)

G2	1 (0.1)	8 (0.4)	87.6 (<0, 98.5)	1 (0.0)	12 (0.4)	91.7 (34.7, 99.0)

G3	1 (0.1)	15 (0.8)	93.4 (49.4, 99.1)	3 (0.1)	20 (0.7)	85.1 (49.6, 95.6)

G4	2 (0.1)	18 (1.0)	89.1 (52.0, 97.5)	2 (0.1)	20 (0.7)	90.1 (57.2, 97.7)

G9^d^	0 (0.0)	14 (0.8)	100 (69.6, 100)	2 (0.1)	25 (0.9)	92.1 (66.1, 98.2)

^a ^Subjects who received a vial where a temperature excursion occurred, subjects who had less than 3 vaccinations or less than 28 days between vaccinations, and subjects who were cross-treated or prematurely unblinded

^b ^In the PP analysis follow-up begins 14 days after dose 3; in the MITT analysis follow-up begins after dose 1

^c ^The numbers in the RV5 and Placebo columns represent the number (rate) of events; the rates reflect the incidence density expressed as the annual number of events per 1000 person-years

^d ^The number of hospitalizations and ED visits among placebo recipients for the per-protocol population has been revised since the NEJM publication. One additional healthcare encounter in the placebo group was identified after the NEJM publication [10]

### Comparison of G1-G4 PP analysis and MITT analysis regardless of serotype stratified by region

Table 5 presents the effect of RV5 on the rates of RVGE healthcare encounters for the G1-G4 PP analysis and the MITT analysis regardless of serotype stratified by region. Based on the G1-G4 PP analysis, use of RV5 resulted in the following reductions in the rate of hospitalizations and ED visits: 94.7% (95% CI: 90.9, 96.9) for Europe, 94.9% (95% CI: 84.0, 98.9) for the US, and 90.0% (95% CI: 29.4, 99.8) for Latin America and the Caribbean. By comparison, use of RV5 resulted in the following reductions in the rate of hospitalizations and ED visits for the MITT analysis regardless of serotype: 92.0% (95% CI: 88.4, 94.6) for Europe, 80.0% (95% CI: 68.7, 87.6) for the US, and 80.2% (95% CI: 28.5, 96.2) for Latin America and the Caribbean. In the US, the difference in the rate reductions for the G1-G4 PP analysis and the MITT analysis regardless of serotype was more pronounced for ED visits. The rate reductions for ED visits were 92.9% (95% CI: 77.4 - 98.6) based on the G1-G4 PP analysis compared with a corresponding 71.6% (95% CI: 54.3 - 82.9) rate reduction based on the MITT analysis regardless of serotype. The rate reductions for hospitalizations were 100% (95% CI: 73.8, 100) based on the G1-G4 PP analysis compared with 97.4% (95% CI: 84.8, 99.9) based on the MITT analysis regardless of serotype.

**Table 5:** Comparison of PP and MITT Analyses for RVGE Healthcare Encounters by Type of Encounter and Region

Region	PP Analysis			MITT Analysis		
	**RV5**	**Placebo**	**% Rate Reduction** **(95% CI)**	**RV5**	**Placebo**	**% Rate Reduction** **(95% CI)**

**Europe**						

*Infants contributing to the analysis of hospitalizations and ED visits*^a^	n = 14018	n = 13984		n = 14831	n = 14734	

Hospitalizations & ED visits^b^	16 (1.7)	301 (32.0)	94.7 (90.9, 96.9)	31 (2.3)	387 (29.4)	92.0 (88.4, 94.6)

Hospitalizations^b^	5 (0.5)	126 (13.4)	96.0 (90.3, 98.4)	14 (1.1)	172 (13.1)	91.9 (85.9, 95.6)

ED visits^b^	11 (1.2)	175 (18.6)	93.7 (87.8, 96.8)	17 (1.3)	215 (16.3)	92.1 (87.0, 95.4)

*Infants contributing to the analysis of office visits in the efficacy substudy from Finland*	n = 1100	n = 1171		n = 1222	n = 1233	

Office visits^c^	7 (4.7)	58 (37.0)	87.2 (67.5, 94.7)	10 (5.3)	59 (30.9)	82.9 (67.0, 92.4)

**United States (including the Navajo and White Mountain Apache Nations)**						

*Infants contributing to the analysis of hospitalizations and ED visits*^a^	n = 12284	n = 12179		n = 15587	n = 15561	

Hospitalizations & ED visits^b^	3 (0.4)	58 (8.0)	94.9 (84.0, 98.9)	24 (1.8)	120 (9.2)	80.0 (68.7, 87.6)

Hospitalizations^b^	0 (0.0)	16 (2.2)	100 (73.8, 100.0)	1 (0.1)	39 (3.0)	97.4 (84.8, 99.9)

ED visits^b^	3 (0.4)	42 (5.8)	92.9 (77.4, 98.6)	23 (1.8)	81 (6.2)	71.6 (54.3, 82.9)

*Infants contributing to the analysis of office visits*	n = 890	n = 925		n = 994	n = 1018	

Office visits^c^	6 (0.8)	40 (53.5)	84.2 (66.2, 95.1)	11 (10.3)	64 (57.4)	82.0 (68.5, 92.2)

**Latin America and the Caribbean (including Puerto Rico)**						

*Infants contributing to the analysis of hospitalizations and ED visits*^a^	n = 2252	n = 2237		n = 2630	n = 2651	

Hospitalizations & ED visits^b^	1 (0.8)	10 (8.0)	90.0 (29.4, 99.8)	3 (1.4)	15 (7.2)	80.2 (28.5, 96.2)

Hospitalizations^b^	1 (0.8)	2 (1.6)	50.2 (<0.0, 99.1)	1 (0.5)	4 (1.9)	75.3 (0, 99.5)

ED visits^b^	0 (0.0)	8 (6.4)	100 (41.2, 100.0)	2 (0.9)	11 (5.3)	82.0 (15.8, 98.0)

^a ^There were 189 infants enrolled in Taiwan who were not included in the regional analyses

^b ^The numbers in the RV5 and Placebo columns represent the number (rate) of events; the rates reflect the incidence density expressed as the annual number of events per 1000 person-years

^c ^Office visits were only documented for a subset of infants from Finland and the US; there were no office visits documented in Latin America and the Caribbean

### Comparison of results for RVGE and AGE health care encounters by intensity of surveillance for PP analysis

Table 6 presents the effect of RV5 on the rates of hospitalizations and ED visits for RVGE and AGE as well as the number and rate of events (annual number of events per 1000 person years) among infants in the RV5 and placebo groups for the PP analysis stratified by the intensity of surveillance at the study site. There were 4,451 infants in the Clinical Efficacy substudy (2,173 RV5; 2,278 Placebo) and the remaining 52,683 infants were participating at other sites (26,473 RV5; 26,210 Placebo - numbers not shown in tables). The relative reduction in the rate of hospitalizations and emergency department visits for RVGE and AGE in the Clinical Efficacy substudy were generally consistent with the rate reductions at other sites. By type of surveillance, the rate reductions were 91.0% (95% CI: 81.7, 95.5) for the G1-G4 PP analysis and 39.7% (95% CI: 28.0, 49.5) for all AGE among parents contacted every 2 weeks compared with 95.9% (95% CI: 92.2, 97.8) for the G1-G4 PP analysis and 41.2% (95% CI: 35.0, 46.8) for all AGE among parents contacted every 6 weeks. However, the rate of G1-G4 RVGE hospitalizations and ED visits reported among infants in the placebo group in the Clinical Efficacy substudy (51.8 per 1000 person years) was more than three times the rate for placebo recipients not in the substudy (15.6 per 1000 person years). The difference in the rates for ED visits was more pronounced than the differences in the rates for hospitalizations. Similarly, the rate of hospitalizations and ED visits for AGE reported among infants in the placebo group in the Clinical Efficacy substudy (131.4 per 1000 person years) was nearly 1.5 times higher than the rate among infants for RVGE in the placebo group for infants not in the Clinical Efficacy substudy (53.8 per 1000 person years).

**Table 6:** PP Analysis for RVGE and AGE Hospitalizations and ED Visits Stratified by Intensity of Surveillance

Type of Health Care Encounter									
**Type of Site**	**Hospitalizations and ED visits**			**Hospitalizations**			**ED visits**		

	**RV5 # (rate)^a ^of events**	**Placebo # (rate)^a ^of events**	**% Rate Reduction** **(95% CI)**	**RV5 # (rate)^a ^of events>**	**Placebo # (rate)^a ^of events**	**% Rate Reduction** **(95% CI)**	**RV5 # (rate)^a ^of events**	**Placebo # (rate)^a ^of events**	**% Rate Reduction** **(95% CI)**

**Sites in the Efficacy Substudy**									

RVGE	10 (4.3)	128 (51.8)	91.0 (81.7, 95.5)	1 (0.4)	27 (10.9)	95.7 (68.5, 99.4)	9 (3.8)	101 (40.9)	89.7 (78.1, 95.1)

AGE	280 (76.2)	483 (131.4)	39.7 (28.0, 49.5)	31 (8.4)	95 (25.8)	66.1 (48.0, 77.8)	249 (67.8)	388 (105.5)	33.2 (18.9, 45.1)

**Sites not in the Efficacy Substudy**									

RVGE	10 (0.6)	241 (15.6)	95.9 (92.2, 97.8)	5 (0.3)	117 (7.6)	95.8 (89.6, 98.3)	5 (0.3)	124 (8.0)	96.0 (90.2, 98.4)

AGE	815 (31.6)	1385 (53.8)	41.2 (35.0, 46.8)	206 (8.0)	480 (18.7)	57.1 (49.0, 64.0)	609 (23.6)	905 (35.2)	32.8 (24.2, 40.4)

^a ^The numbers in the RV5 and Placebo columns represent the number (rate) of events; the rates reflect the incidence density expressed as the annual number of events per 1000 person years

## Discussion

The results for REST presented here demonstrate that the reduction in the rates of hospitalizations and ED visits associated with the use of RV5 remained very high in the MITT analysis regardless of serotype despite small differences between the G1-G4 PP analysis and the MITT analysis regardless of serotype. The MITT analysis was more inclusive than the PP analysis because it included protocol violators, and RVGE episodes regardless of serotype immediately following dose 1. Of the 69,274 randomized infants enrolled in REST, only 3,096 (4.5%) were excluded from the MITT analysis of whom 1481 were in the vaccine group and 1615 were in the placebo group. Among those excluded who received at least one dose of vaccine, the majority did not have stool specimens collected to determine whether the episodes were attributable to RVGE or they did not have evaluable follow-up time. The small differences between the G1-G4 PP analysis and the MITT analysis regardless of serotype for the overall study population appeared to be associated with the inclusion of protocol violators in the MITT analysis and the time needed for an immune response to develop within the first 14 days after dose 1. There was no difference in the reduction in the rate of hospitalizations and ED visits for the G1-G4 PP analysis and the PP analysis regardless of serotype. High efficacy has been established with post hoc analyses from 14 days after dose 1 until 14 days after dose 3 [27].

The consistency of the results for the PP and MITT analyses extended to other subgroups within the study population. The comparison of the PP and MITT analyses for premature infants has already been published [28]. The reduction in the rate of hospitalizations and ED visits was 100% (95% CI: 74.0, 100) for the G1-G4 PP analysis and 95.5% (95% CI: 76.4, 99.9) for the MITT analysis regardless of serotype among premature infants [28].

The higher number of healthcare encounters included in the MITT analysis provided better precision for the reductions in hospitalizations and ED visits combined for each of the most prevalent serotypes circulating in the US, Europe, and Latin America, and the broader scope of the MITT analysis enhanced the generalizability of such results. The rate reduction for the G2 serotype based on the MITT analysis also provided better sensitivity to the difference between RV5 and placebo for G2.

When the analyses were stratified by region, the rates of healthcare utilization among infants in the placebo group differed across geographic areas, which may be explained by differences in healthcare systems. However, the relative effect of RV5 on reducing these rates was generally consistent across regions based on the PP analysis [20]. The rate reductions for the U.S. and the Latin American/Caribbean regions based on the MITT analysis regardless of serotype were somewhat lower than the G1-G4 PP analyses. In the US the difference in the rate reductions for the G1-G4 PP analysis and the MITT analysis regardless of serotype was more pronounced for ED visits. The rate reductions for hospitalizations were 100% (95% CI: 73.8, 100) based on the G1-G4 PP analysis compared with 97.4% (95% CI: 84.8, 99.9) based on the MITT analysis regardless of serotype.

When the study population was stratified based on the intensity of surveillance, the rates of healthcare encounters among infants in the placebo group differed but the impact of the vaccine on reducing the rates of healthcare encounters was consistent for infants included in the Clinical Efficacy substudy compared with other infants. The difference in the rates of hospitalizations and ED visits for RVGE based on the intensity of surveillance likely reflects a combination of better recall of events among parents of infants who were monitored more closely as well as the higher proportion of stool specimens collected. The efficacy of RV5 against RVGE of any severity was also evaluated in the Clinical Efficacy substudy. These results were reported in an earlier publication for the PP and MITT analyses based on the G1-G4 serotypes [10,27]. In the first rotavirus season after vaccination the efficacy against G1-G4 RVGE regardless of severity was 74% (95% CI: 66.8, 79.9) and 60% (95% CI: 57.5, 67.1) for the PP and MITT analyses respectively [10]. When all RVGE regardless of serotype were included, the efficacy was 71.8% (95% CI: 64.5, 77.8 - data not shown in tables) and 50.9% (95% CI: 41.6, 58.9 - data not shown in tables) for the PP and MITT analyses respectively.

## Conclusion

Our analyses demonstrate the robust and consistent performance of the vaccine and its relevance for infants and young children in a prelicensure study. These findings reflect the generalizability of the effect of RV5 on reducing RVGE including the healthcare burden associated with serotypes not included in the vaccine and was evident when follow-up began immediately following the first dose. Furthermore, the results did not depend on the location of the sites or the intensity of the surveillance. Parents of children in the Clinical Efficacy substudy were contacted more frequently compared to other parents and were more likely to recall events. This increased the power of the study to detect differences between the vaccine and the placebo groups with a smaller sample size but it did not influence the observed relative effect of RV5 on reducing the healthcare burden. This observation should reassure researchers designing future clinical trials for rotavirus gastroenteritis that the time interval chosen to collect healthcare utilization data should not bias the results but it may affect the power of the study.

## Competing interests

Dr. David Matson, Dr. Leif Gothefors, and Dr. Pierre Van Damme have been investigators for Merck. Dr. Koch has served as the principal investigator for a cooperative agreement between Merck Research Laboratories and the University of North Carolina at Chapel Hill. Although this agreement was discontinued by Merck Research Laboratories a few years ago, Dr. Koch's initial activity with respect to the matters in this manuscript were through this agreement. Dr. Robbin Itzler and Dr. Mark DiNubile are full-time employees of Merck Research Laboratories (as indicated on the title page) and may own stock and/or stock options in the company. Dr. Penny Heaton was an employee of Merck & Co., Inc., at the time the study was conducted.

REST and derivative analyses were sponsored and funded by Merck & Co., Inc., which markets RV5 under the brand name RotaTeq^®^. Authors had access to all study data upon request. This report was principally drafted by Dr. Itzler with substantive input from all co-authors, and subsequently approved by each author in its essentially final form. The paper underwent formal review by the sponsor.

## Authors' contributions

RI participated in the conception and design of the study for the healthcare utilization endpoints as well as analysis and interpretation of data and preparation of the manuscript.

GK helped with the design of the statistical methods for the healthcare utilization endpoints and provided a critical review of the manuscript. DM, LG, and PVD participated in the enrollment of subjects, collection of data, and critical review of the manuscript. MD helped with the analysis and interpretation of the data as well as preparation of the manuscript. PH was involved in all phases of the study including study concept and design, collection as well as analysis and interpretation of the data, and critical review of manuscript. All of the authors read and approved the final manuscript.

## Pre-publication history

The pre-publication history for this paper can be accessed here:

http://www.biomedcentral.com/1471-2431/10/42/prepub
